# Spectroscopic (FT-IR, FT-Raman, ^1^H- and ^13^C-NMR), Theoretical and Microbiological Study of *trans o*-Coumaric Acid and Alkali Metal *o*-Coumarates

**DOI:** 10.3390/molecules20023146

**Published:** 2015-02-13

**Authors:** Małgorzata Kowczyk-Sadowy, Renata Świsłocka, Hanna Lewandowska, Jolanta Piekut, Włodzimierz Lewandowski

**Affiliations:** 1 Division of Chemistry, Bialystok University of Technology, Wiejska 45E, Bialystok 15-351, Poland; E-Mails: m.kowczyk@pb.edu.pl (M.K.-S.); r.swislocka@pb.edu.pl (R.S.); j.piekut@pb.edu.pl (J.P.); 2 Institute of Nuclear Chemistry and Technology, 16 Dorodna St., Warsaw 03-195, Poland; E-Mail: h.lewandowska@ichtj.waw.pl

**Keywords:** 2-hydroxycinnamic acid, alkali metal *o*-coumarates, FT-IR, FT-Raman, NMR, antimicrobial activity

## Abstract

This work is a continuation of research on a correlation between the molecular structure and electronic charge distribution of phenolic compounds and their biological activity. The influence of lithium, sodium, potassium, rubidium and cesium cations on the electronic system of *trans o*-coumaric (2-hydroxy-cinnamic) acid was studied. We investigated the relationship between the molecular structure of the tested compounds and their antimicrobial activity. Complementary molecular spectroscopic techniques such as infrared (FT-IR), Raman (FT-Raman), ultraviolet-visible (UV-VIS) and nuclear magnetic resonance (^1^H- and ^13^C-NMR) were applied. Structures of the molecules were optimized and their structural characteristics were calculated by the density functional theory (DFT) using the B3LYP method with 6-311++G** as a basis set. Geometric and magnetic aromaticity indices, atomic charges, dipole moments and energies were also calculated. Theoretical parameters were compared to the experimental characteristics of investigated compounds. Correlations between certain vibrational bands and some metal parameters, such as electronegativity, ionization energy, atomic and ionic radius, were found. The microbial activity of studied compounds was tested against *Escherichia coli*, *Bacillus subtilis*, *Pseudomonas aeruginosa*, *Staphylococcus aureus*, *Proteus vulgaris* and *Candida albicans*.

## 1. Introduction

Phenolic acids (PAs) have varied biological activity in the human body. They help to scavenge free radicals, chelate metal ions, induce changes in enzyme activity and protein availability. It was also confirmed that PAs prevent coronary heart disease, cancer, inflammation and diabetes. Hydroxycinnamic acid and its derivatives (e.g., coumaric, caffeic, ferulic and sinapic acids) are important pharmaceuticals for high blood pressure, stroke prevention and possess antitumor activity [1,2,3].

There are three isomers of coumaric acid, *i.e*. *o*-, *m*- and *p*-coumaric acid, that differ in the position of the hydroxyl group substitution on the phenyl group. In Nature the most widespread is the *para*-isomer [4]. Coumaric acids can be found in cereals (barley, rye, corn, oats, rice, wheat), fruits (berries, grapes, apples, currants), legumes (beans, peas), nuts (hazelnut, pecan, peanut, walnut), vegetables (celery, tomato, garlic, carotene), oilseeds (flax, mustard), red wine, beer and tea [3,5,6].

Phenolic acids are responsible for the sour and bitter taste of some food products of plant origin. It was found that the characteristic flavor of products made from flour obtained from maize embryos is the result of the presence of certain PAs, namely: ferulic and *o*-and *p*-coumaric acids [7]. Coumaric acid shows lower antioxidant activity than ferulic acid. This is related to the presence of only one hydroxyl group in coumaric acid. In order to increase the antioxidant activity compared to the low-density lipoprotein (LDL) fraction it should be subjected to esterification with tartaric acid. Several papers report on the antioxidative properties of coumaric acids [8,9]. Moreover, cinnamic acid and its derivatives: *p*-coumaric, ferulic and sinapic acid, are important copolymer building blocks of lignin [10].

Cinnamic acid is a precursor for lignin biosynthesis, being transformed into 4-coumaric acid (4-hydroxycinnamic acid), ferulic acid (4-hydroxy-3-methoxycinnamic acid), and sinapic acid (4-hydroxy-3,5-dimethoxycinnamic acid) before its incorporation into a lignin polymer.

Pannala *et al*. [11] showed that hydroxycinnamates can interact with peroxynitrite depending on the nature of the ring substitutions and the positions of the hydroxyl groups. Studies on the potency of PAs to inhibit tyrosine nitration by peroxynitrite demonstrated that PAs’ activity decreases in the order: caffeic acid→chlorogenic acid→ferulic acid→*p*-coumaric acid→*o*-coumaric acid→*m*-coumaric acid. *p*-Coumaric acid inhibits LDL peroxidation, is shown to be antimutagenic, antigenotoxic, and antimicrobial, inhibits cellular melanogenesis, plays a role in immune regulation in humans and reduces the risk of stomach cancer [8]. This acid is an instance of the PAs commonly used as additives in chemical, food, health, cosmetic and pharmaceutical industries.

In our earlier works, the influence of various metals and halogens on the electronic structure of the benzoic, salicylic, nicotinic and isonicotinic acids were studied [12,13,14,15]. Those ligands were treated as models to study enzymes and other biologically important molecules. We studied the relationship between certain parameters (*i.e*., ionic potential, electronegativity, atomic mass of the metal or ring substituent) and electronic charge distribution in the whole molecule. In case of those ligands the decrease in the ionic potential of the metal brings about the decrease in the uniform charge distribution in the aromatic ring. In the case of halogens, the ionic potential was even more important than the polarity of the C-X bond. The question arises then if the same or other parameters influence the electronic charge distribution in the case of alkali metal *o*-coumarates.

The second aim of this work is to continue earlier research [16,17,18,19] on a correlation between the molecular structure and electronic charge distribution of phenolic compounds and their biological activity. In one of our papers [20] the influence of lithium, sodium, potassium, rubidium and cesium on the electronic system of the *p*-coumaric (4-hydroxycinnamic) acid was investigated. Experimental and theoretical FT-IR, FT-Raman, ^1^H- and ^13^C-NMR spectra of *p*-coumaric acid and its salts were registered and analyzed.

In the present paper another isomer *trans o*-coumaric acid and its lithium, sodium, potassium, rubidium and cesium complexes were synthesized and studied by spectroscopic methods (FT-IR, FT-Raman, UV-VIS, ^1^H- and ^13^C-NMR). A logical series of metals was selected, (different radius but the same degree of oxidation). The alkali metals taken to this study meet the following criteria (which are important from the point of view of further possible applications): (1) possibility of practical application because of the good solubility of the alkali metal compounds in water and polar solvents; (2) as small as possible harmfulness to the human body and the natural environment; (3) availability, stability and ease of preparation.

UV-VIS spectroscopy was applied to study/establish the changes in π→π* transition energies and draw conclusions on delocalization energy changes. The antimicrobial activity of the studied compounds was tested against *Escherichia coli*, *Pseudomonas aeruginosa*, *Staphylococcus aureus*, *Proteus vulgaris*, *Bacillus subtilis* and, *Candida albicans*, which allowed us to draw some correlations between physicochemical parameters and microbial properties of *trans o*-coumaric acid and alkali metal *o*-coumarates. Elementary analysis for all synthesized complexes was performed in order to confirm the chemical composition of the tested compounds. The theoretically predicted values were compared with the experimentally measured data and the results were also discussed. Optimized geometrical structures, atomic charges, infrared and NMR spectra of *trans o*-coumaric acid and Li, Na and K *trans o*-coumarates were calculated by the B3LYP/6-311++G** method.

## 2. Results and Discussion 

### 2.1. Calculated Geometrical Structure

**Figure 1:**
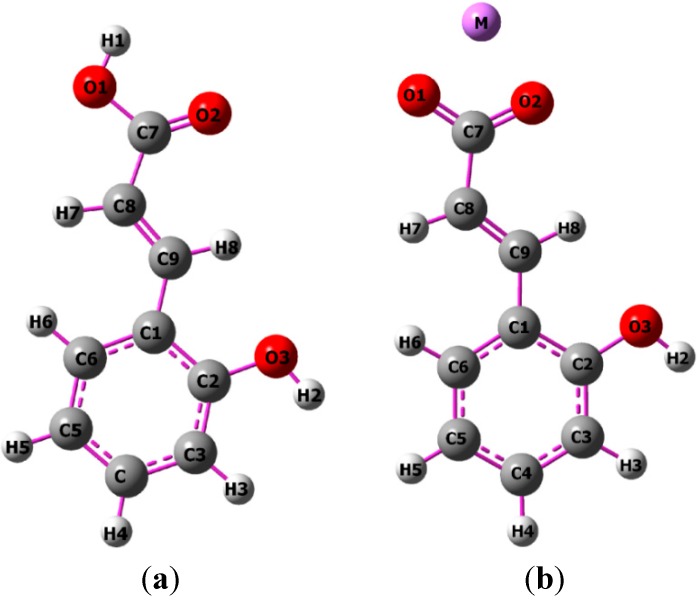
Structures of: *trans o*-coumaric acid (**a**) and alkali metal *o*-coumarates (M = Li, Na, K, Rb, Cs) (**b**).

Optimized geometrical structures were calculated using B3LYP/6-311++G** quantum chemical method [21]. The distances between atoms in *trans o*-coumaric acid and alkali metal *o*-coumarate molecules and the angles between bonds were calculated and the values are presented in Table 1. The atom numbering is shown in Figure 1. Changes in the bond distances and angles between bonds suggest that alkali metal cations influence the molecular structure of *trans o*-coumaric acid. The bond lengths of C1-C9, C7-C8, C7-O1 and C7-O2 do not change in the series of salts, although they are different from the corresponding values calculated for the 2-hydroxycinnamic acid molecule. Large differences between bonds are also noticed for C7-O2 (it increases by: 0.065 Å (Li); 0.056 Å (Na); 0.058 Å (K)). A slightly lower extension of the C7-C8 bond between carbon atoms of the carboxylic acid and C1-C9 is observed. The bond lengths of C7-O1 in the acid is higher than in the salts (0.006–0.014 Å). The values of the bond lengths in aromatic ring are almost the same in acid and salts.

**Table 1:** Distances between atoms (Å), bonds angles (°) and geometric aromaticity indices calculated for *trans o*-coumaric acid and lithium, sodium and potassium *o*-coumarates.

	Acid		Li	Na	K
	Calculated	Experimental [22]	Calculated		
**Distances [Å]**					
**C1-C2 ^a^**	1.412	1.401	1.411	1.412	1.411
**C2-C3**	1.396	1.389	1.396	1.397	1.396
**C3-C4**	1.389	1.368	1.390	1.391	1.391
**C4-C5**	1.396	1.379	1.396	1.397	1.395
**C5-C6**	1.387	1.379	1.388	1.389	1.389
**C6-C1**	1.406	1.394	1.405	1.407	1.405
**C1-C9**	1.460	1.466	1.464	1.466	1.466
**C7-C8**	1.471	1.460	1.481	1.343	1.497
**C8-C9**	1.344	1.337	1.343	1.501	1.341
**C2-O3**	1.365	1.353	1.368	1.369	1.370
**C7-O1**	1.364	1.329	1.278	1.270	1.272
**C7-O2**	1.211	1.229	1.276	1.266	1.268
**O3-H2**	0.963	0.790	0.963	0.965	0.962
**C3-H3**	1.086	0.904	1.086	1.086	1.087
**C4-H4**	1.084	0.989	1.084	1.084	1.084
**C5-H5**	1.083	0.917	1.083	1.083	1.084
**C6-H6**	1.083	1.082	1.083	1.083	1.083
**C8-H7**	1.083	1.059	1.084	1.085	1.085
**C9-H8**	1.085	0.613	1.084	1.084	1.084
**O1-H1(M)**	0.968	0.987	1.854	2.372	2.516
**O2-Me**	-	-	1.850	2.365	2.509
**Angles [°]**					
**C1-C2-C3**	120.867	120.590	121.014	121.063	121.145
**C2-C3-C4**	120.181	119.880	120.253	120.152	120.338
**C3-C4-C5**	120.066	120.410	119.881	119.962	119.699
**C4-C5-C6**	119.522	120.310	119.565	119.578	119.609
**C5-C6-C1**	121.962	120.590	122.110	122.015	122.262
**C6-C1-C2**	117.402	118.210	117.177	117.230	116.947
**C2-C1-C9**	119.370	118.660	119.606	119.585	119.781
**C1-C9-C8**	126.973	126.160	126.882	127.421	126.824
**C7-C8-C9**	120.355	120.130	121.977	122.953	122.615
**C8-C7-O1**	111.123	112.460	118.273	116.528	116.496
**C8-C7-O2**	126.998	126.060	121.069	119.740	119.511
**C7-C8-H7**	116.222	116.610	114.692	114.930	114.494
**C9-C8-H7**	123.423	123.250	123.332	122.117	122.891
**C8-C9-H8**	116.851	117.230	116.776	116.648	116.691
**C1-C9-H8**	116.176	86.400	116.342	115.931	116.486
**C9-C1-C2**	119.370	118.660	119.606	119.585	119.781
**C1-C2-O3**	119.370	116.780	117.705	117.807	117.759
**C3-C2-O3**	119.370	122.620	121.281	121.130	121.096
**C3-C4-H4**	119.370	118.660	119.661	119.595	119.736
**C4-C5-H5**	119.370	125.840	120.326	120.338	120.331
**C6-C5-H5**	119.370	113.680	120.109	120.085	120.060
**C5-C6-H6**	119.370	118.640	119.100	119.048	119.119
**C1-C6-H6**	119.370	120.600	118.790	118.937	118.619
**C6-C1-C9**	119.370	123.130	123.217	123.185	123.272
**C9-C8-C7**	120.355	120.130	121.977	122.953	122.615
**C5-C4-H4**	120.344	120.930	120.458	120.443	120.566
**C2-O3-H2**	110.021	114.110	109.702	109.812	109.442
**C7-O1-H1(M)**	106.701	110.930	82.753	89.771	91.302
**O1-C7-O2**	121.879	121.480	120.657	123.732	123.994
**C7-O2-M**	-	-	82.988	90.165	91.693
**O1-M-O2**	-	-	73.601	56.332	53.011
**C1-C9-C8-C7**	−179.998	−179.210	179.998	−179.999	179.998
**C6-C1-C9-C8**	0.011	7.290	−0.023	−0.030	−0.023
**C9-C8-C7-O1**	−179.999	176.670	179.999	179.994	179.999
**C9-C8-C7-O2**	0.002	−2.790	0.004	−0.004	0.004

^a^: Atom numbering.

In the case of angles, the increase in the order: acid < Li *<* Na *<* K was observed for C1-C2-C3 angles in the aromatic ring. The highest changes were noticed for the carboxylate group, as the C7-O1-H1(M) angle decreases by 23.948° (Li); 16.930° (Na); 15.399° (K). C8-C7-O1 angle increases by 7.150° (Li); 5.405° (Na); 5.372° (K), while the C8-C7-O2 angle decreases by 5.928° (Li); 7.257° (Na); 7.487° (K). In the aromatic ring only small angle changes were observed.

Dipole moments, energies and geometric aromaticity indices [23,24] for *trans o*-coumaric acid and *o*-coumarates were calculated and are presented in Table 2. Comparing values of dipole moment for molecules of lithium, sodium and potassium salts of 2-hydroxycinnamic acid an increasing tendency can be seen along the series: Li (1.909 D) < H (5.046 D) < Na (5.488 D) < K (6.155 D). The energy values decrease in the series: H→Li→K→Na. The obtained results show that the degree of ionic bonding increases from H to K atom, because of an increase in symmetrization of the carboxylate ion and in the aromaticity of the molecule.

**Table 2:** The calculated aromaticity indices, dipole moments (Debye) and total energy (Hartree, 1 hartree = 2625.5 kJ/mol) calculated using B3LYP/6-311++G** method for *trans o*-coumaric acid and *o*-coumarates lithium, sodium and potassium.

	Acid	Li	Na	K
Geometric Aromaticity Indices				
A_j_	0.991	0.992	0.992	0.993
BAC	0.900	0.910	0.906	0.915
HOMA	0.955	0.958	0.952	0.960
GEO	0.020	0.017	0.018	0.016
EN	0.025	0.025	0.030	0.025
I_6_	92.652	93.182	93.023	93.482
Dipole Moment				
Debye (D)	5.046	1.909	5.488	6.155
Energy				
Hartree	−573.632	−580.615	−735.372	−1173.020

Aj—normalized function of the bond variance lengths; BAC—Bond Alternation Coefficient; HOMA—Harmonic Oscillator Model of Aromaticity; (GEO: bond length alternation; EN: bond elongation) I_6_—Bird’s index were calculated.

Geometric aromaticity indices: A_j_—normalized function of the bond variance lengths; BAC—Bond Alternation Coefficient HOMA—Harmonic Oscillator Model of Aromaticity; and I_6_—Bird’s index were calculated. These index values calculated for salts are almost the same as those obtained for *o*-coumaric acid. Comparing the aromaticity indices for *trans o-*coumaric acid with those for *p*-coumaric acid (HOMA = 0.963; A_j_ = 0.992; BAC = 0.887; I_6_ = 93.134) [20] and cinnamic acid (HOMA = 0.968; A_j_ = 0.995; BAC = 0.915; I_6_ = 94.430) [25] show that the aromaticity of these compounds increases in the series: *trans o*-coumaric acid→*p*-coumaric acid→cinnamic acid (except BAC).

Mulliken, APT (atomic polar tensor) and NBO (natural bond orbital) atomic charges on the atoms of 2-hydroxycinnamic acid molecule and its alkali metal salts are gathered in Table 3. The electron density increases around atoms C1 and C8 and at the same time decreases around atoms C2, C4, C6 and C9 in the following order: K *o*-coumarate > Na *o*-coumarate > Li *o*-coumarate *> trans o*-coumaric acid, according to APT and NBO calculations.

**Table 3:** The atomic charges for *trans* o-coumaric acid as well as for lithium, sodium and potassium o-coumarates calculated in the B3LYP/6-311++G6** level.

Atom Position	*Trans o*-Coumaric Acid			Li *o-*Coumarate			Na *o-*Coumarate			K *o-*Coumarate		
	Mulliken	APT	NBO	Mulliken	APT	NBO	Mulliken	APT	NBO	Mulliken	APT	NBO
**C1 ^a^**	1.790	−0.230	−0.141	1.920	−0.146	−0.131	1.855	−0.074	−0.123	1.855	−0.058	−0.121
**C2**	0.148	0.513	0.353	0.007	0.481	0.345	−0.110	0.454	0.340	−0.099	0.450	0.338
**C3**	−0.358	−0.115	−0.208	−0.296	−0.101	−0.282	−0.249	−0.088	−0.282	−0.272	−0.087	−0.283
**C4**	−0.278	0.055	−0.165	−0.257	0.026	−0.175	−0.271	0.002	−0.183	−0.254	−0.002	−0.185
**C5**	−0.610	−0.212	−0.228	−0.673	−0.187	−0.230	−0.597	−0.166	−0.231	−0.602	−0.162	−0.231
**C6**	−1.334	0.072	−0.144	−1.307	0.046	−0.150	−1.350	0.024	−0.155	−1.352	0.019	−0.156
**C7**	0.043	1.575	0.761	−0.446	1.498	0.735	0.218	1.418	0.738	0.295	1.448	0.743
**C8**	−0.239	−0.542	−0.307	0.530	−0.449	−0.275	−0.162	−0.336	−0.262	−0.495	−0.338	−0.260
**C9**	−0.036	0.370	−0.100	−0.010	0.248	−0.130	0.059	0.134	−0.157	−0.039	0.116	−0.160
**H1 (Me)**	0.292	0.307	0.481	0.151	0.843	0.886	0.557	0.884	0.918	0.997	0.957	0.939
**H2**	0.265	0.294	0.472	0.260	0.286	0.469	0.257	0.280	0.467	0.256	0.279	0.466
**H3**	0.126	0.028	0.203	0.116	0.022	0.200	0.117	0.019	0.197	0.115	0.017	0.197
**H4**	0.163	0.041	0.208	0.161	0.036	0.205	0.152	0.032	0.203	0.151	0.031	0.203
**H5**	0.182	0.037	0.209	0.177	0.032	0.207	0.171	0.028	0.204	0.170	0.027	0.204
**H6**	0.083	0.049	0.205	0.085	0.049	0.205	0.079	0.049	0.205	0.077	0.048	0.205
**H7**	0.209	0.051	0.208	0.186	0.037	0.200	0.171	0.023	0.192	0.173	0.024	0.192
**H8**	0.245	0.086	0.238	0.264	0.086	0.237	0.253	0.084	0.235	0.252	0.085	0.235
**O1**	−0.185	−0.856	−0.691	−0.334	−1.121	−0.817	−0.463	−1.098	−0.813	−0.502	−1.141	−0.822
**O2**	−0.310	−0.871	−0.611	−0.339	−1.036	−0.827	−0.490	−1.019	−0.820	−0.527	−1.064	−0.830
**O3**	−0.195	−0.649	−0.67	−0.193	−0.649	−0.672	−0.198	−0.649	−0.674	−0.199	−0.648	−0.674
**Sum: Ring**	−0.642	0.083	−0.605	−0.606	0.119	−0.623	−0.722	0.152	−0.634	−0.724	0.160	−0.638
**C=C**	−0.275	−0.172	−0.407	0.520	−0.201	−0.405	−0.103	−0.202	−0.419	−0.534	−0.222	−0.420
**COO^−^**	−0.452	−0.152	−0.541	−1.119	−0.659	−0.909	−0.735	−0.699	−0.895	−0.734	−0.757	−0.909

^a^: Atom numbering.

**Figure 2:**
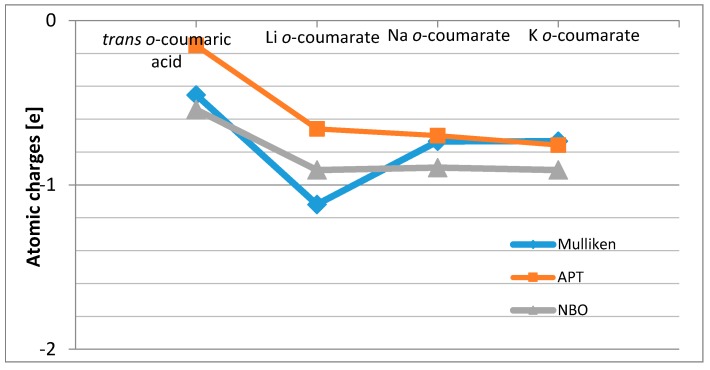
The changes in Mulliken, APT and NBO total charges on carboxylate group in alkali metal *o-*coumarate molecules in comparison with *trans o-*coumaric acid.

The highest changes of total charge independently on the method used were observed on carboxylate anion (Figure 2).The theoretical wavenumbers of IR, Raman and chemical shifts in NMR spectra were obtained and compared with experimental spectra.

### 2.2. NMR Spectra

**Figure 3:**
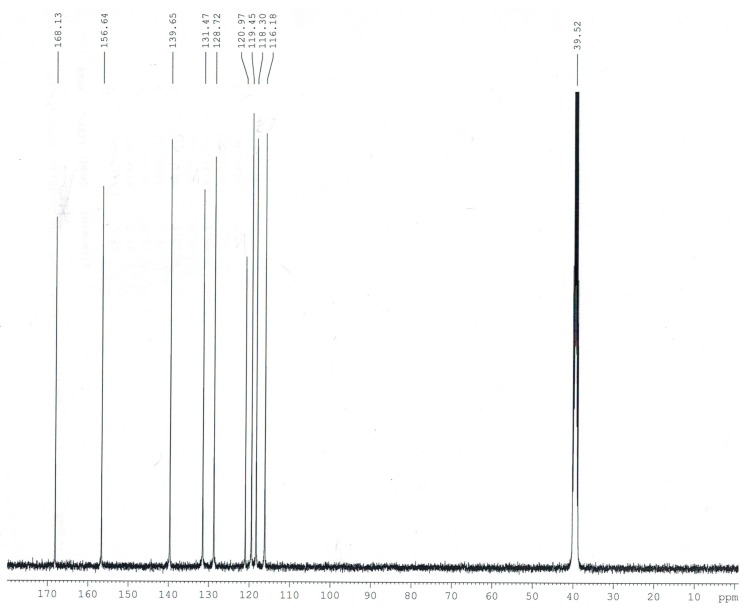
Experimental ^13^C-NMR spectrum of *trans o-*coumaric acid.

**Figure 4:**
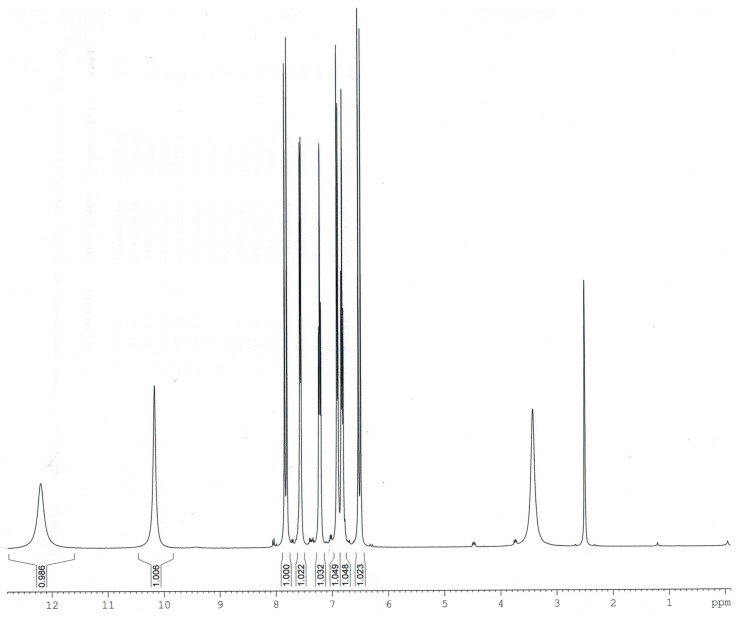
Experimental ^1^H-NMR spectrum of *trans o-*coumaric acid.

**Figure 5:**
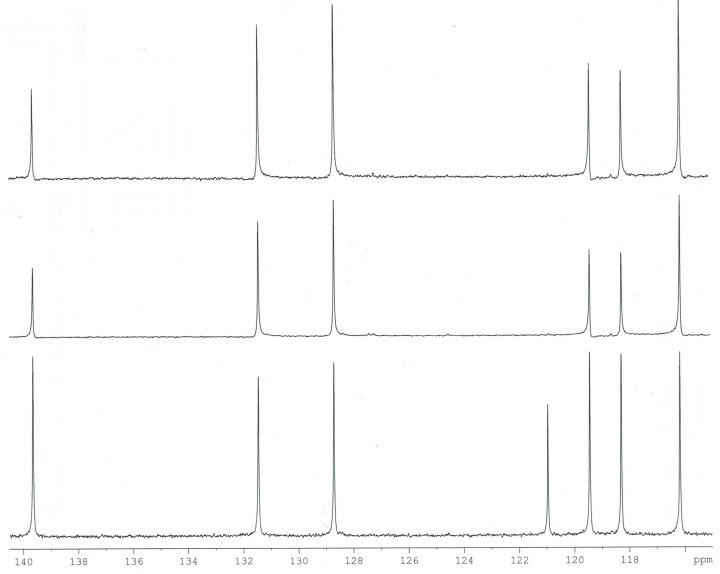
Experimental DEPT NMR spectrum of *trans o-*coumaric acid.

Experimental and calculated (B3LYP/6-311++G**) data for the chemical shifts in ^1^H- and ^13^C-NMR spectra obtained for *trans o*-coumaric acid and *o-*coumarates are gathered in Table 4. The numbering of atoms is shown in Figure 1. The experimental ^1^H-, ^13^C- and DEPT NMR spectra are shown in Figure 3, Figure 4 and Figure 5. Only experimental data were obtained for rubidium and cesium *o-*coumarates. A good correlation between experimental and calculated chemical shifts is obtained. Values of correlation coefficient R for carbon (^13^C-NMR) are in the range of 0.9742 to 0.9918. For proton (^1^H-NMR) the corresponding range is 0.9456–0.9695.

In the proton spectra of *o-*coumarates the signals from almost all protons are shifted downfield in comparison to appropriate signals in the spectrum of *trans o*-coumaric acid (except H3 and H7). This tendency suggests that introduction of metal cations causes the decrease in ring current intensity. Moreover the general tendency can be observed in the decrease of chemical shifts of particular protons in the series: Li→Na→K→Rb→Cs.

This order depicts the increase in the destabilization of the electronic system. In the carbon spectra of *o*-coumarates there is almost no regularity in the chemical shifts of carbons along the series of alkali metal *o*-coumarates. Only the values of chemical shifts of C5 and C6 atoms regularly increase in the series: acid→Li→Na→K→Rb→Cs.

**Figure 6:**
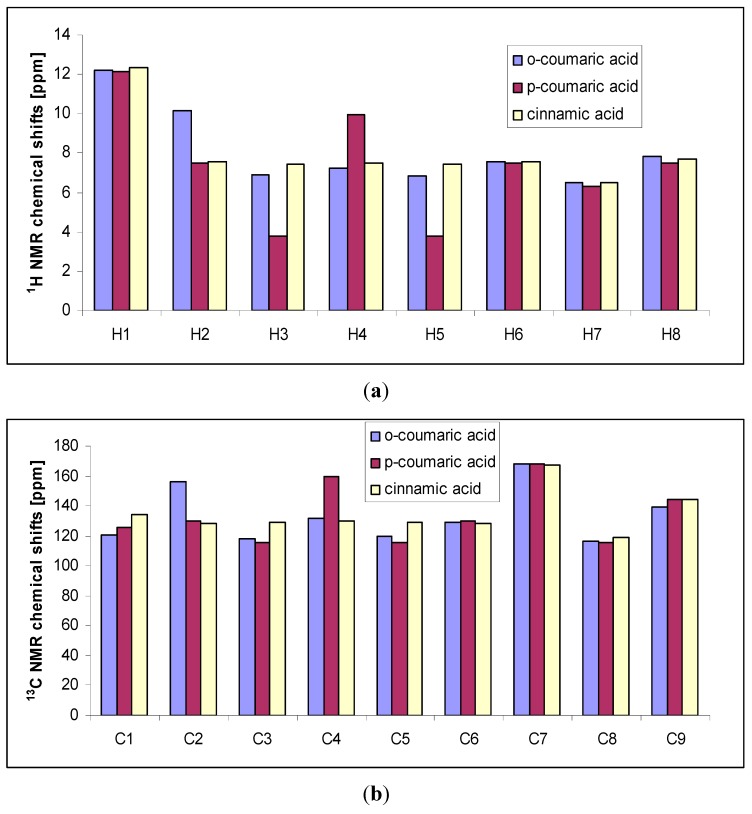
Experimental ^1^H- (**a**) and ^13^C-NMR (**a**) chemical shifts of *trans o-*coumaric acid, *p-*coumaric acid and cinnamic acid.

Chemical shift values of signals for *trans o*-coumaric acid were compared with those for *p*-coumaric acid [20] and cinnamic acid [25], and presented in Figure 6. The signals from protons No. 2, 8 and carbon No. 2 are shifted downfield in comparison to the appropriate signals in the spectrum of *trans o*-coumaric acid, whereas the signals from proton No. 4 and carbons No. 1 and 9 are shifted upfield. The values of chemical shifts of H1, H6, H7, H8, C6, C7, C8 and C9 were found almost the same.

**Table 4:** Experimental and calculated (B3LYP/6-311++G**) chemical shifts [ppm] in ^1^H- and ^13^C-NMR spectra of DMSO solution of *trans o*-coumaric acid and *o-*coumarates.

Atoms	*transo*-Coumaric acid		Li *o*-Coumarate		Na *o*-Coumarate		K *o*-Coumarate		Rb *o*-Coumarate	Cs *o*-Coumarate
	Exp.	Theoret.	Exp.	Theoret.	Exp.	Theoret.	Exp.	Theoret.	Exp.	Exp.
H1	12.20	6.03	-	-	-	-	-	-	-	-
H2	10.17	4.94	-	4.77	-	4.77	-	4.68	-	-
H3	6.90	7.00	6.93	6.98	6.91	6.97	6.95	6.98	6.96	6.95
H4	7.21	7.58	7.06	7.47	7.05	7.43	7.02	7.45	7.01	6.99
H5	6.82	7.20	6.71	7.15	6.71	7.13	6.67	7.12	6.65	6.60
H6	7.56	8.01	7.39	8.09	7.36	8.06	7.33	8.05	7.32	7.29
H7	6.51	6.76	6.50	6.74	6.49	6.73	6.54	6.74	6.53	6.55
H8	7.82	8.82	7.62	8.59	7.51	8.32	7.49	8.39	7.48	7.45
C1	120.97	126.79	127.16	128.21	127.39	129.24	127.28	129.28	127.38	127.45
C2	156.64	165.17	156.6	163.87	156.59	163.38	157.04	163.24	157.42	157.56
C3	118.30	121.59	118.56	121.28	118.42	121.34	117.83	121.13	117.71	117.58
C4	131.47	141.22	129.30	138.69	128.96	137.51	128.58	137.35	128.67	128.45
C5	119.45	127.09	123.07	126.68	123.25	126.96	123.41	126.74	123.46	123.57
C6	128.72	134.14	127.22	133.25	127.81	132.76	128.17	132.58	128.37	128. 49
C7	168.13	175.50	172.18	191.67	171.99	184.30	171.24	185.76	171.31	171.19
C8	116.18	117.72	116.31	127.45	116.28	130.99	116.37	132.13	116.49	116.52
C9	139.65	150.54	133.48	143.54	132.75	140.77	132.51	140.22	132.61	132.48

**Figure 7:**
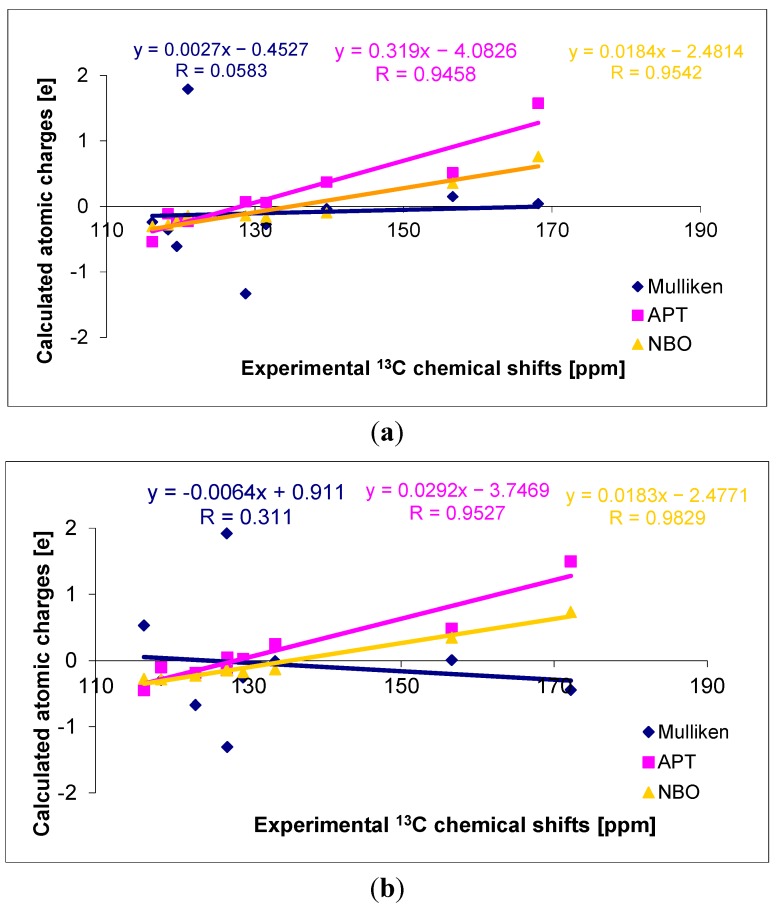
The correlation between the calculated atomic charges on carbon atoms in *trans o*-coumaric acid and (**a**) and Na *o*-coumarate (**b**) molecules and experimental ^13^C-NMR chemical shifts.

The relationship between calculated atomic charges spectra and experimental chemical shifts from the carbon for *trans o*-coumaric acid and *o-*coumarates was found and can be seen in Figure 7. Namely, the increase of the atomic charge along with the increase of the chemical shift is observed. The highest correlation coefficients R were obtained for the linear correlation between the chemical shifts and the NBO (for acid: 0.9542; for Li salt: 0.9866; for Na salt: 0.9829; for K salt: 0.9806) and APT (for acid: 0.9458; for Li salt: 0.9527; for Na salt: 0.9555; for K salt: 0.9477) atomic charges. Whereas the lower ones were obtained for the Mulliken atomic charges (for acid: 0.0583; for Li salt: 0.1311; for Na salt: 0.1284; for K salt: 0.1934).

### 2.3. Vibrational Spectra

**Figure 8:**
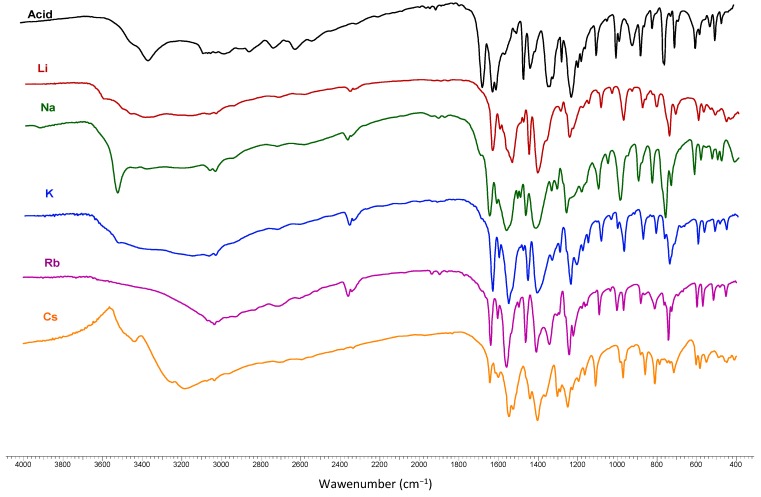
The IR spectra of *trans o*-coumaric acid and lithium, sodium, potassium, rubidium and cesium *o*-coumarates.

The IR spectra of *trans o*-coumaric acid and lithium, sodium, potassium, rubidium and cesium *o*-coumarates are presented in Figure 8. The wavenumbers, intensities and assignments of bands occurring in the FT-IR and FT-Raman spectra of *trans o*-coumaric acid and the synthesized lithium, sodium, potassium, rubidium and cesium *o-*coumarates are presented in Table 5 and Table 6. Theoretical wavenumbers of the bands in vibrational spectra and also band intensities were obtained using B3LYP/6-311++G** method. The numeration of the normal vibrations of benzene ring was done according to the notation used by Varsányi [26]. A good correlation between experimental and theoretical wavenumbers in the IR spectra of *trans o*-coumaric acid as well as its salts were obtained; the correlation coefficients R for acid equal 0.9972; the corresponding value for lithium salt is 0.9976; for sodium salt −0.9975 and for potassium salt 0.9973.

While comparing the spectra obtained for alkali metal *o*-coumarates with that obtained for acid, there can be observed a disappearance of bands characteristic for stretching vibrations of carboxyl group νOH: 2847 cm^−1^ (IR), 3771 cm^−1^ (theoret.), νC=O: 1668 cm^−1^ (IR), 1665 cm^−1^ (Raman), 1781 cm^−1^ (theoret.) and deformation vibrations βOH: 1460 cm^−1^ (IR), 1446 cm^−1^ (Raman), 1279 cm^−1^ (theoret.). The medium intensity bands connected with carboxylic group also disappeared, namely νC-OH: 1169 cm^−1^ (IR), 1170 cm^−1^ (Raman), γCO (698 cm^−1^ and 688 cm^−1^ in IR and Raman, respectively).

Besides there are other bands characteristic only for spectra of salts concerning asymmetric and symmetric stretching vibrations ν_as_COO^−^ and ν_s_COO^−^; in plane deformations β_as_COO^−^ and β_s_COO^−^ and out-of-plane bending γ_s_COO^−^ of carboxylate group. The very strong bands assigned to the stretching vibrations of the carboxylate group are located in the range 1543–1380 cm^−1^ (IR), 1563–1377 cm^−1^ (Raman).

**Table 5:** The wavenumbers (cm^−1^) and assignments of bands occurring in the experimental FT-IR, FT-Raman and calculated spectra of *trans o*-coumaric acid.

FT-IR				FT-Raman		Assignment	No [26]
Exp.		Theoret.	Int.	Exp.			
3356	s ^a^	3837	85.64			ν ^b^(OH)_ar_	
		3771	108.64			ν(OH)	
		3200	13.48			ν(CH)_ar_, ν(CH)_C=C_	
		3195	0.84			ν(CH)_ar_	2
3080	m	3185	9.85	3061	vw	ν(CH)_ar_, ν(CH)_C=C_	20a
3055	m	3183	2.73	3045	vw	ν(CH)_ar_, ν(CH)_C=C_	20b
2972	m	3175	2.59			ν(CH)_ar_, ν(CH)_C=C_	7b
		3152	12.85			ν(CH)_ar_	
2847–2616	m					ν(OH)	
1668	s	1781	372.28	1665	vw	ν(C=O)	
1616	vs.	1673	302.05	1627	m	ν(CC)C=C	
1599	vs.	1621	8.71	1604	vs	ν(CC)_ar_	8b
1557	m	1644	94.51			ν(CC)_ar_	8a
1497	m	1488	66.5	1509	w	ν(CC)_ar_	19b
1460	s	1529	11.43			ν(CC)_ar_	19a
1427	s	1377	193.31	1446	w	β(OH), β(CH)_ar_	
1337	s	1326	31.15	1333	vw	β(CH)C=C, β(CH)_ar_	3
1327	s	1359	35.02			β(CH)_C=C_, β(CH)_ar_	14
1267	s	1351	28.97	1266	w	β(CH)_C=C_	
		1279	11.61			β(OH)	
1219	vs	1274	62.45	1225	s	ν(C-OH)_ar_	
		1225	13.72			βCH	
1186	s	1191	73.89			β(CH)_ar_	9a
1169	s	1131	639.08	1170	w	ν(C-OH)	
1150	sh	1108	49.47	1151	w	β(CH)_ar_	
1094	m	1184	6.15	1097	vw	β(CH)_ar_	18b
1040	w	1065	5.58	1039	w	β(CH)_ar_	18a
	m	1037	28.63	996	vw	γ(CH)_C=C_	
993		967	10.48			ν(CCO)	
978	m	977	0.18	981	vw	γ(CH)_ar_,	5
		895	20.88			γ(CH)_C=C_	
910	m					γ(OH)	
868	m	849	0.09	871	vw	γ(CH)_ar_	17b
851	w			852	vw	γ(CH)_ar_	
810	w			809	w	γ(CH)	
754	s	810	12.21	758	vw	γ(CH)_ar_	10b
748	s					φ(CCC)	4
698	m	769	49.02	688	w	γ(C=O)	
		758	46.43			γ(CH)	11
681	w	709	10.9			α(CCC)	
592	m			595	vw	β(CH)	
571	m			563	vw	α(CCC)	6a
519	w	658	10.99	511	vw	β(CO), α(CCC)	6b
		598	43.27			β(CH)	
494	m	462	0.92			φ(CC)	16a
		582	89.8			γ(OH)	
		514	5.43			α(CCC)	
		460	19.93			β(CH)	9b
461	w			458	vw	φ(CC)	16b

^a^ s—strong; m—medium; w—weak; v—very; sh—shoulder; ^b^ The symbol “ν” denotes stretching vibrations. “β” denotes in-plane bending modes. “γ” designates out-of-plane bending modes; “φ(CCC)” denotes the aromatic ring out-of-plane bending modes; “α(CCC)” designates the aromatic ring in-plane bending modes.

**Table 6:** The wavenumbers (cm^*−*1^) and assignments of bands from the FT-IR and FT-Raman spectra of lithium, sodium, potassium, rubidium and cesium *o*-coumarates.

Li *o*-Coumarate						Na *o*-Coumarate					K *o*-Coumarate							Rb *o*-Coumarate				Cs *o*-Coumarate				Assignment	No. [26]
FT-IR				FT-Raman		FT-IR				FT-Raman	FT-IR					FT-Raman		FT-IR		FT-Raman		FT-IR		FT-Raman			
Exp.		Theoret.	Int.	Exp.		Exp.		Theoret.	Int.	Exp.	Exp.			Theoret.	Int.	Exp.		Exp.		Exp.		Exp.		Exp.			
3564	W ^a^	3839	75.44			3526	w	3811	69.42			3519	w	3840	66.91							3433	w			ν ^b^(OH)_ar_	
		3197	15.93					3195	18.22					3194	18.26											ν(CH)_ar_, ν(CH)_C=C_	
3154	w	3191	0.15					3188	0.24				w	3189	0.3											ν(CH)_ar_	2
3073	w	3186	8.64	3073	w	3071	w	3184	13.28	3070	vw	3070	w	3183	12.34	3075	vw	3071	w	3074	vw	3058	w	3061	m	ν(CH)_ar_, ν(CH)_C=C_	20a
3040	w			3038	vw	3038	w			3045	w	3035	w			3042	w	3036	w	3037	w	3035	w			ν(CH)_ar_, ν(CH)_C=C_	20b
2949	w	3172	4.45					3173	2.87					3171	3.09			2932	w					2932	vw	ν(CH)_ar_, ν(CH)_C=C_	7b
		3179	7.74					3165	13.65					3167	12.54											ν(CH)_C=C_	
		3148	15.71					3153	15.89					3143	18.84											ν(CH)_ar_	
1641	vs	1681	162.62			1638	vs	1678	101.79	1639	vs			1683	88.66	1642	vs	1639	vs	1640	vs	1631	vs	1627	vs	ν(CC)_C=C_	
1605	m	1621	14.18	1645	vs	1605	m	1617	8.41	1608	m	1641	vs	1620	11.72	1608	m	1605	m	1607	m	1605	m	1605	vs	ν(CC)_ar_	8b
		1643	34.17	1607	s			1638	22.02			1605	m	1641	19.49											ν(CC)_ar_	8a
1543	vs	1527	403.69	1546	vw	1557	vs	1568	460.22	1560	w	1557	vs	1559	411.25	1563	m	1560	vs	1555	w	1552	vs			ν_as_(COO^−^)	
1485	m	1486	89.69			1483	w	1483	71.48	1470	vw	1483	w	1484	71.48	1499	w	1499	w	1500	vw	1470	w			ν(CC)_ar_	19b
1458	vs	1530	63.41	1462	w	1460	vs	1525	30.82			1460	vs	1528	36.01	1469	vw	1464	vs	1467	vw	1456	s	1454	w	ν(CC)_ar_	19a
1414	vs	1430	700.96	1429	w	1412	vs	1398	600.77	1427	w	1412	vs	1399	708.7	1416	w	1410	vs	1409	w	1380	vs	1377	w	ν_s_(COO^−^)	
1345	sh	1324	19.83			1339	m	1321	22.08	1318	m	1339	w	1320	23.45	1342	vw	1344	s	1347	vw					β(CH)_C=C_, β(CH)_ar_	3
		1360	36.02					1358	40.64					1359	35.57											β(CH)_C=C_, β(CH)_ar_	14
1298	m	1347	29.19	1290	sh	1298	m	1345	24.84			1298	w	1344	21.84			1303	sh	1308	w	1301	m			β(CH)_C=C_	
1254	vs	1276	16.74	1256	s	1242	vs	1275	18.2	1243	s	1241		1271	20.04	1241	s	1244	vs	1245	s	1248	s	1263	m	β(CH)_C=C_	
1234	sh	1267	127.71			1213	s	1265	96.92	1215	w	1221		1265	95.72			1223	m			1218	w			ν(C-OH)_ar_	
		1220	6.36					1218	12.07					1217	8.34											βCH_ar_	
1184	w	1191	57.63			1182	m	1193	56.94			1178		1190	61.43	1181	vw	1180	vw							β(CH)_ar_	9a
1155	w	1106	50.27	1162	m	1155	m	1106	45.17	1156	m	1163		1105	48.44	1156	w	1163	vw	1165	w	1150	m	1158	w	β(CH)_ar_	
1094	s	1183	10	1097	vw	1090	s	1180	8.24	1094	vw	1090		1181	7.46	1093	vw	1092	m	1094	vw	1093	m			β(CH)_ar_	18b
1040	m	1066	2.65	1041	m	1040	vw	1062	3.7	1042	m	1035	w	1065	4.24	1038	m	1035	vw	1038	m	1040	w	1041	w	β(CH)_ar_	18a
						1007	w					1010	w			1011	w	1003	m	1002	w						
980	s	973	0.16	984	m	974	s	971	0.05	976	vw	969	s	971	0.12	970	w	968	m	970	vw	970	s			γ(CH)_ar_,	5
937	w	1034	32.89			922	vw	1033	35.16					1032	35.34											γ(CH)_C=C_	
		846	0.08					841	0.01					843	0.08											γ(CH)_ar_	17b
883	m			887	w	878	m			879	m	879	m			881	vw	881	m	884	vw	881	m			β_s_(COO^−^)	
814	s			818	w	812	m			815	w	811	m			814	w	812	m	814	w	813	m			γ(CH)_C=C_	
		826	27.14					820	18.8					819	22.26											γ(CH)_ar_	10b
						770	w	726	18.4	772	vw	763	w	726	38.13	764	vw									γ_s_(COO^−^)	
748	vs			764	w	743	s			730	w	741	s					743	vs			751	vs			φ(CCC)	4
718	s	756	68.5	720	w			752	70.9					753	69.87							712	w			γ(CH)	11
669	vw	705	0.4			689	vw	703	0.07			695	w	703	0.03			669	vw							α(CCC)	
602	s			601	vw	600	m			603	w	597	m			600	w	598	m	599	w	596	m			β_as_(COO^−^)	
575	m					570	s			572	vw	569	s			572	vw	569	m	570	vw	565	w			α(CCC)	6a
517	w	631	178.97	513	vw	515	m	610	21.82	516	vw	515	w	612	28.6	517	w	515	m	516	vw	515	w			α(CCC)	6b
		466	9.93			490	w	467	9.95			486	w	466	8.74	488	w	482	vw	484	vw					φ(CC)	16a
		517	0.36					512	5.44					514	7.96											α(CCC)	
460	m			458	vw	455	m					450	m			452	w	451	w	454	w	461	w			φ (CC)	16b

^a^ s—strong; m—medium; w—weak; v—very; sh—shoulder; ^b^ The symbol “ν” denotes stretching vibrations. “β” denotes in-plane bending modes. “γ” designates out-of-plane bending modes; “φ(CCC)” denotes the aromatic ring out-of-plane bending modes; “α(CCC)” designates the aromatic ring in-plane bending modes.

These band are sensitive to the type of metal coordination and degree of complex hydration. β_as_COO^−^ and β_s_COO^−^ are in the range 883–596 cm^−1^ (IR), 887–596 cm^−1^(Raman) and γ_s_COO^−^ modes of are at ~770 cm^−1^ (IR), (Raman). The difference between the wavenumbers of asymmetric and symmetric stretching of the carboxylic anion vibrations (ΔνCOO^−^) increased in the IR and Raman spectra from lithium to cesium *o*-coumarates. The increase in this value means the increase in the ionic character of metal-oxygen bond. The values of ∆βCOO^−^ were also calculated and increased in the IR spectra along the series: Na→Li→K→Rb→Cs (278, 281, 282, 283, 285 cm^−1^) in Raman the order is: K→Na→Rb→Li (276, 281, 285, 286 cm^−1^).

Correlations between the chosen wavenumbers of vibrational bands in the IR spectra of *o*-coumarates and some alkali metal parameters, such as electronegativity, atomic and ionic radii, affinity, energy of ionization and ionic potential were analyzed. The best correlation was obtained for ionic radius (R = 0.9888) and ionization energy (R = 0.9687) for deformation band of carboxylate group (β_as_COO^−^). The analysis of these data shows that the ionic radius and ionization energy are the most important metal parameters in view of influence of the metal on the vibration structure of *o*-coumarates.

In the spectra of *trans o*-coumaric acid and its metal salts bands from the hydroxyl group attached to the aromatic ring are present: (1) in the spectrum of the acid: νOH_ar_ 3356 cm^−1^ (IR), 3837 cm^−1^ (theoret.); νC-OH_ar_ 1219 cm^−1^ (IR), 1225 cm^−1^ (Raman), 1274 cm^−1^ (theoret._._); (2) in the spectra of salts: νOH_ar_ 3564-3433 cm^−1^ (IR), 3840–3811 cm^−1^ (theoret._._); νC-OH_ar_ 1234–1213 cm^−1^ (IR), 1215 cm^−1^ (Raman), 1267–1265 cm^−1^ (theoret.). Bands are located in the range 3154–2932 cm^−1^ (IR), 3074–2932 cm^−1^ (Raman) 3200–3143 cm^−1^ (theoret.), can be assigned to the CH stretching modes of aromatic ring and the double bond between C8 and C9 atoms. A peak around 1640 cm^−1^ (IR and Raman), 1680 cm^−1^ (theoret.) derives from the CC stretching vibrations of the double bond. The bands of the β(CH) vibrations are located in the range 1345–1035 cm^*−*1^ (IR), 1333–1038 cm^*−*1^ (Raman) and 1326–1062 cm^*−*1^ (theoret.). The bands at 980–712 cm^*−*1^ (IR), 984–720 cm^*−*1^ (Raman) and 1037–752 cm^*−*1^ (theoret.) are assigned to out-of-plane bending vibrations γCH. The wavenumbers of aromatic bands numbered as 20a, 20b, 7b, 19a, 9a, 5, 6b, 16a and 16b decrease in comparison to free acid, while the wavenumbers of 8b and 3 bands in the spectra increase.

### 2.4. UV Spectra

The UV spectra of *trans o*-coumaric acid and lithium, sodium, potassium, rubidium and cesium *o*-coumarates were recorded. The maxima of π→π* bands for *trans o*-coumaric acid were located at 210, 272, and 314 nm. In the UV spectra of *o*-coumarates the bands were shifted toward lower wavelengths, with the maxima at 210, 268, 312 nm respectively, similarly for all the salts. The noted hypsochromic shifts indicate a decrease in the delocalization energy and therefore, a perturbation effect of alkali metals on the electronic system of ligand.

### 2.5. Microbial Activity

**Figure 9:**
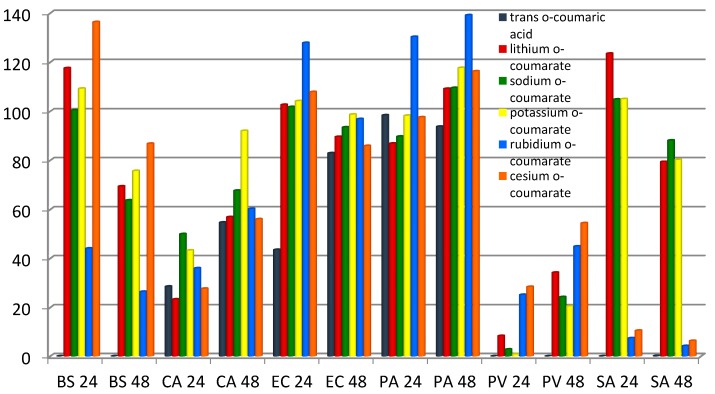
The degree of growth inhibition induced by the studied compounds in *Baccillus ssubtilis* (BS), *Candida albicans* (CA), *Escherichia coli* (EC), *Pseudomonas aeruginosa* (PA), *Proteus vulgaris* (PV) and *Staphylococus aureus* (SA); evaluated after 24 and 48 h of incubation.

Evaluation of the antimicrobial activity of *trans o*-coumaric acid and lithium, sodium, potassium, rubidium and cesium *o*-coumarates was measured as a degree of growth inhibition or stimulation in individual bacteria or fungi cultures, in relation to the control (non-treated) sample and depicted as a percent of control. The average values given in Figure 9 were calculated from the data obtained in the four experiments taken for each preparation and strain. The results obtained after 24 and 48 h of incubation are shown in Figure 9.

*Trans o-*coumaric acid induced a considerable growth inhibition in *Bacillus subtilis*, *Proteus vulgaris* and *Staphylococcus aureus* after 24 h and 48 h of treatment. The studied compounds strongly inhibited the growth of the bacterium *Proteus vulgaris* after 24 h of incubation. Lithium, sodium and potassium salts support the growth of *Bacillus subtilis, Staphylococcus aureus* and *Escherichia coli.* All salts showed a stimulating effect after 48 hours incubation in relation to bacteria *Pseudomonas aeruginosa.* Potassium and rubidium salts inhibited the growth of *Escherichia coli* to the lowest degree. Strong inhibitory effects of cesium and rubidium *o*-coumarates shown relative to *Staphylococcus aureus* (~95%).

**Figure 10:**
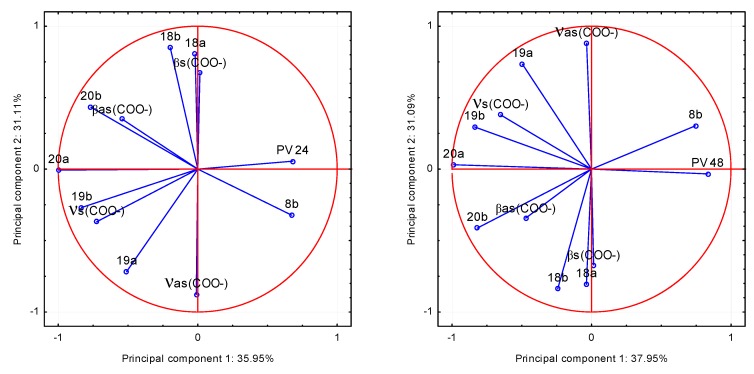
The values of the band wavenumbers in the FT–IR spectra of *trans o*-coumaric acid and alkali metal *o*-coumarates in relation to the degree of growth inhibition of the bacteria *Proteus vulgaris* (PV) after 24 and 48 h of incubation in the two principal components system.

**Figure 11:**
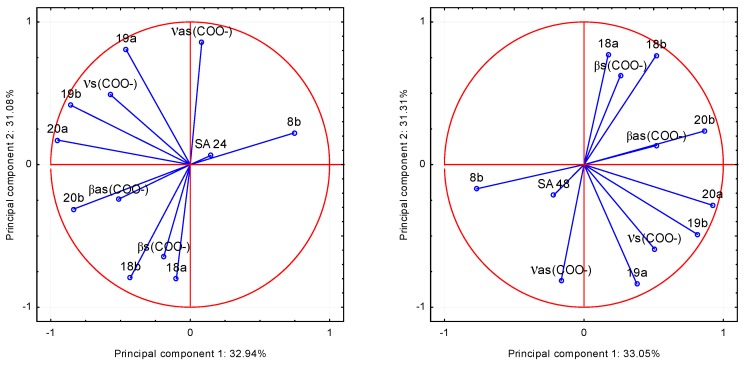
The values of the band wavenumbers in the FT–IR spectra of *trans o*-coumaric acid and alkali metal *o*-coumarates in relation to the degree of inhibition of growth in *Staphylococcus aureus* (SA) after 24 and 48 h of incubation in the two principal components system.

The correlation between the physicochemical parameters and microbial properties of *trans o-*coumaric acid and alkali metal *o*-coumarates was examined. On the basis of the spectroscopic data: the values of wavenumbers of infrared spectra of the compounds and the parameters describing their antimicrobial activity the principal component analysis (PCA) was performed. This method is most commonly used for multivariate exploratory analysis of experimental data. Analysis was made on the basis of the factor loadings values being correlation coefficients describing the correlation between the antimicrobial properties of the compounds and the wavenumbers of selected bands in FT-IR spectra. Based on the screen chart, two main components were selected to data analysis. To investigate which of the bands described by the wavenumbers, are strongly positively or negatively correlated with the microbiological data, the projection weights on the plane defined by pairs of main factors was made. Correlations are determined by the angle between the two weight vectors, which initial point and the ends are defined by respective weight values. The relationship between the input variables and the main components is presented as a graph of configuration of points representing the variables in the system of the first two principal components (Figure 10 and Figure 11). The coordinates of these points are corresponding coefficients of variables, in this case, the wavenumbers of selected bands in the FT-IR spectra. The more given variable is closer to the edge of the wheel, the better is its representation of the main components defining the coordinate system, *i.e.*, the greater part of the information contained in the variable is carried by the main components.

Analysis of the data leads to the conclusion that the most statistically significant correlation was demonstrated between the degree of inhibition of bacterial growth: *Proteus vulgaris* (PV) and *Staphylococcus aureus* (SA) and the wavenumbers of the following bands in the spectra of the studied compounds: positive: 20b, β_as_(COO^−^) and negative: 8b, both after 24 and 48 h of incubation. It shows that the FT-IR spectra can be a good source of data for the quantitative analysis of the relationship between the molecular structure of the compound and its biological activity. This finding strongly points to the fact, that the analysis of the physicochemical structures can bring significant prerequisites on the biological activity of compounds. The presented method and analysis can be found useful in design of the syntheses of microbial active analogues.

## 3. Experimental Section

### 3.1. General Information

*Trans o-*coumaric acid was purchased from Sigma-Aldrich (Saint Louis, MO, USA) with a stated purity 97% and was used as such without further purification. Lithium, sodium, potassium, rubidium and cesium *o*-coumarates were prepared by dissolving *trans o*-coumaric acid powder in a water solution of the appropriate alkali metal hydroxide in a stoichiometric ratio of 1:1. The mixed solution was slowly condensed at 70 °C, then,the solvent was removed by drying at 105 °C. The obtained salts were anhydrous, as indicated by a lack of bands characteristic of crystallization water in the corresponding FT-IR spectra.

### 3.2. Spectroscopic Methods

The FT-IR spectra were recorded with an Equinox 55 FT-IR spectrometer Bruker, (Billerica, MA, USA) within the range of 4000–400 cm^*−*1^. The resolution of spectrometer was 1 cm^*−*1^. Samples in the solid state were measured in KBr matrix pellets obtained with hydraulic press, under 739 MPa pressure. FT-Raman spectra of solid samples in capillary tubes were recorded in the range of 4000–400 cm^−1^ with a FT-Raman accessory of the Perkin Elmer (Waltham, MA, USA) System 2000. The ^1^H (400.15 MHz), ^13^C (100.63 MHz) spectra were measured on a Bruker Avance II 400 spectrometer in DMSO-*d*_6_ solution at 25°C. Chemical shifts are reported in ppm on δ scale and referenced to the solvent resonances (2.50 ppm for ^1^H and 39.52 ppm for ^13^C). The UV spectra in water solution were recorded on a HACH-LANGE (Düsseldorf, Germany) spectrophotometer between 190 and 400 nm. The compounds were studied in aqueous solutions with concentrations 5 × 10^−5^ mol/dm^3^.The crystal structure of *o*-coumaric acid was found in the Crystallographic Structural Data Base (CSD) crystallographic database and visualized with the Mercury 1.4.2 program [22].

### 3.3. Quantum-Mechanical Calculations

Calculations of optimized geometrical structures of *trans o*-coumaric acid and its alkali metal salts were performed using the GAUSSIAN 09 package of programs [21] running on a PC computer.

### 3.4. Microbiological Tests

Five species of bacteria: *Escherichia coli* (PCM 2268), *Bacillus subtilis* (PCM 2021), *Proteus vulgaris* (PCM 2269), *Pseudomonas aeruginosa* (PCM 2270), *Staphylococcus aureus* (PCM 2267) and one species of fungi *Candida albicans* (PCM 2566) were used for antimicrobial tests. Bacterial cultures where purchased from Polish Collection of Microorganisms (PCM, Wroclaw, Poland) .The investigated microorganisms were inoculated on broth medium and stored in 35 °C (for fungi in 25 °C) for 24 h. Solutions of tested compounds were prepared by dissolving 0.2 g of each of them in 9.8 mL of deionized water. The concentration of compounds in the culture broth was 0.1%. The growth of tested cells was monitored using turbidimetry by measuring the optical density at 600 nm on JASCO spectrophotometer. The microbiological tests for investigated compounds were carried out in deionized water medium. The samples were incubated in 35 °C for bacteria and 25 °C for fungi. The number of colonies was directly proportional to optical density, which was estimated after 24 h and 48 h incubation.

### 3.5. Elementary Analysis

The elementary analysis for all synthesized complexes were performed. The complexes were anhydrous. The results of elementary analysis are as follows: for lithium *o*-coumarate: %C = 60.42:60.49 (calc. %C = 60.36), %H = 4.29:4.33 (calc. %H = 4.22); for sodium *o*-coumarate: %C = 56.57:56.75 (calc. %C= 58.07), %H = 3.67:3.70 (calc. %H = 3.62); for potassium *o*-coumarate: %C= 53.30:53.31 (calc. %C= 53.49), %H = 3.54:3.60 (calc. %H = 3.49); for rubidium *o*-coumarate: %C= 42.99:43.03 (calc. %C= 43.48), %H = 2.95:3.03 (calc. %H = 2.84); for cesium *o*-coumarate: %C= 36.99:37.03 (calc. %C= 36.51), %H = 2.55:2.63 (calc. %H = 2.38).

## 4. Conclusions

In this paper the influence of lithium, sodium, potassium, rubidium and cesium cations on the electronic system of *trans o*-coumaric acid was studied by means of FT-IR, FT-Raman, UV-VIS, ^1^H- and ^13^C-NMR, *ab initio* calculations and microbiological tests. Substitution of hydrogen with alkali metals ions in the carboxylic group brought about some characteristic changes in the molecular spectra as well as in the geometrical structures of the investigated molecules. The experimental parameters were compared to calculated characteristics of studied compounds and they indicate good linear correlations.

The calculated aromaticity indices (A_j_, BAC and I_6_–Bird’s index) for *trans o*-coumaric acid were almost the same as those of all salts. The aromaticity increased in the series: *trans o*-coumaric acid→ *p*-coumaric acid→cinnamic acid, what suggests that the compounds studied here in possess less aromatic properties than *p*-coumaric acid and cinnamic acid. In the case of salts the same relationship is observed. Theoretical studies of the geometrical parameters have shown that the substitution of the metal in the carboxyl group causes a slight change in the geometry of the aromatic ring. Geometric parameters (almost the same values of bond lengths and angles in the ring for all the salts, independently on the metal ion also suggest that *p-*coumaric acid has a higher aromaticity. Perturbation of the electronic system of molecules increases along the series: acid→Li→Na→K.

Chemical shift changes of were also noted in the proton and carbon NMR spectra. The chemical shifts of all protons decreased in the series Li→Na→K→Rb→Cs and the chemical shifts of carbon no. 5 and 6 increased in the same series. The highest correlation coefficients were obtained between the experimental data and the theoretical ones calculated by the NBO and APT methods.

The intensity and wavenumbers in the case of bands No. 20a, 20b, 7b, 19a, 9a, 5, 6b, 16a and 16b decrease in comparison to the free acid, which points to a perturbation in the electron charge distribution of the ligand. Ionic radius and ionization energy are the most important metal parameters in view of the influence of the metal on the vibration structure of *o*-coumarates. In the case of benzoates and *o*-coumarates the Δν_COO−_ values increase in the series: lithium→sodium→potassium→rubidium→cesium (in FT-IR and FT-Raman spectra). In the case of salicylates and *p*-coumarates these values scatter [27]. The increase in the value of Δν_COO−_ means the increase in the ionic character of metal-oxygen bond. In addition, the value of Δν_COO−_ for *p*-coumaric acid was higher than *o*-coumaric acid (in FT-IR, FT-Raman and theoretical spectra). In the UV-VIS spectra of the studied compounds, the hipsochromic shifts indicate that the alkali metal cations perturb the electronic system of the molecule.

After 24 and 48 h of incubation, *trans o-*coumaric completely inhibited growth of the bacteria: *Bacillus subtilis*, *Proteus vulgaris* and *Staphylococcus aureus.* Based on the principal component analysis, it was found that the most statistically significant correlations exist between the degree of inhibition of bacterial growth *Proteus vulgaris* and *Staphylococcus aureus* and the wavenumbers of 20b, β_as_(COO^−^) and 8b bands in the spectra of studied compounds.

The electronic properties of substituent have a significant effect on the degree of ionization and the polarity of chemical compounds, which influences their ability to penetrate cell membranes and the strength of binding to receptors. Therefore it is very important to be able to measure the electronic parameters of molecules and connect them with the corresponding biological properties. In addition to the physicochemical parameters (dipole moments, hydrogen bonding, conformation and distances between the atoms) discussed above, there are others which also play a role in molecular modeling. The use of these parameters is limited, because of the difficulty in measuring them. However, the biological activity of most compounds is a result of many physicochemical parameters.
